# Association between serum adiponectin and HbA1c is independent of the severity of clinical presentation in young adults with new onset type 1 diabetes

**DOI:** 10.1007/s40618-016-0458-0

**Published:** 2016-03-25

**Authors:** S. Pilacinski, B. Wierusz-Wysocka, D. Zozulinska-Ziolkiewicz

**Affiliations:** Department of Internal Medicine and Diabetology, Poznan University of Medical Sciences, Raszeja Hospital, Mickiewicza 2, 60-834 Poznan, Poland

**Keywords:** Type 1 diabetes, Adiponectin, Hemoglobin A, glycosylated, Diabetic ketoacidosis, Weight loss

## Background and aims

Serum adiponectin concentration is higher in patients with type 1 diabetes (T1DM) when compared with healthy, BMI-matched controls [1]. Hyperadiponectinemia was found to be associated with chronic hyperglycemia in established T1DM and with catabolic state in other medical conditions, as anorexia nervosa [2].

It is unknown whether the association between hyperglycemia and high adiponectin level in T1DM may be attributed to the catabolic state present in decompensated diabetes.

Also, there is no available evidence on the factors associated with serum adiponectin at diagnosis of the disease, when hypeglycaemia and catabolic state often coexist. Therefore, we evaluated the determinants of serum adiponectin concentration in patients with newly diagnosed T1DM.

## Patients and methods

We included 120 consecutive patients (40 women and 80 men, aged 18–35 years, mean age 25 ± 5 years) hospitalized with newly diagnosed autoimmune type 1 diabetes. The diagnosis was confirmed by positive assay for at least one islet autoantibody: to glutamic acid decarboxylase 65 (anti-GAD) or to insulinoma-associated antigen (IA2-Ab) or islet cell antibodies (ICA). Among these, 23 patients (19 %) presented with diabetic ketoacidosis (DKA), defined according to the ADA 2009 criteria. Informed consent was obtained from all individual participants included in the study. Clinical and biochemical characteristics of the study group are shown in Table 1.

**Table 1:** Characteristics of 120 adults at diagnosis of type 1 diabetes

Parameter	Value
Sex, *n* (M/F)	80/40
Age (years)	25 ± 5
Body mass index (kg/m^2^)	22.1 ± 3.2
Systolic blood pressure (mmHg)	117 ± 9
Diastolic blood pressure (mmHg)	76 ± 7
Glycaemia (mmol/l)	23.8 ± 8.8
C-peptide (μg/l)	0.82 (0.55–1.17)
HbA1c (%)	10.8 ± 2.3
Total cholesterol (mmol/l)	4.45 ± 1.33
LDL-cholesterol (mmol/l)	2.92 ± 1.05
HDL-cholesterol (mmol/l)	1.17 ± 0.29
Triglycerides (mmol/l)	1.15 (0.89–1.57)
hsCRP (mg/l)	0.77 (0.4–2.20)
Adiponectin (µg/ml)	5.10 (3.44–7.82)
Diabetic ketoacidosis, *n* (%)	23 (19 %)
Duration of symptoms before diagnosis (days)	28 (14–45)
Weight loss before diagnosis (kg)	6 (3–9)
Relative weight loss before diagnosis (% initial weight)	8.5 (3.8–13.2)
Current cigarette smokers, *n* (%)	50 (42 %)

*hsCRP* serum C-reactive protein concentration measured with highly sensitive method; Means ± SD, Median (IQR) or *n* (%)

At diagnosis of diabetes, anthropometric measurements were taken and data on the duration of clinical symptoms, body weight before onset of symptoms and smoking status were collected using a questionnaire. Serum adiponectin was measured using ELISA kit (Quantikine, R&D). HbA_1c_ was measured using high-performance liquid chromatography (normal reference range 4.1–6.4 %). Serum C-peptide concentration was determined using immunoenzymatical method (IMMULITE, DPC), normal range: 1.1–5.0 ng/ml. In statistical analysis (Statistica 10, StatSoft Inc., Tulsa, USA) Student’s *t* test or Mann–Whitney *U* test, as appropriate, were used to compare serum adiponectin concentration between subgroups. Pearson or Spearman correlation coefficients between continuous variables and serum adiponectin concentration was calculated. In the multivariate linear regression model we determined the association between serum adiponectin concentration and HbA1c, with adjustment for sex and variables that reflect catabolism and inflammation.

## Results

Serum adiponectin concentration at diagnosis was higher in women than in men (mean ± SD: 8.07 ± 4.31 vs. 5.41 ± 3.17 µg/ml, *p* < 0.001) and in patients diagnosed with DKA than in other patients (8.75 ± 4.29 vs. 5.77 ± 3.45 µg/ml, *p* < 0.001). Serum adiponectin concentration was also higher in patients positive for IA2-Ab than in IA2-Ab-negative subjects [6.32 (IQR: 4.15–8.69) vs. 4.31 (IQR: 2.89–7.38) µg/ml, *p* = 0.034] and in subjects positive for three autoantibodies than in those positive only for one or two antibody specificities [6.87 (IQR: 4.37–8.65) vs. 4.28 (IQR: 2.94–7.49) µg/ml, *p* = 0.018]. Adiponectin level correlated positively with HbA1c (*r* = 0.39, *p* < 0.001) and with relative weight loss before diagnosis (*r* = 0.20, *p* = 0.03), and negatively with BMI (*r* = −0.36, *p* < 0.001), systolic (*r* = −0.25, *p* = 0.005) and diastolic (*r* = −0.28, *p* = 0.002) blood pressure. Adiponectin concentration was negatively associated with baseline serum C-peptide concentration in men, but not in women (Spearman’s *R*: −0,35, *p* = 0.002). Adiponectin level was not associated with plasma glucose, serum cholesterol (C), LDL-C, HDL-C and triglyceride concentration. In multivariate linear regression model, the association between adiponectin level and HbA1c remained significant after adjustment for age, sex, BMI, presence of DKA, and relative weight loss before diagnosis of diabetes [*R*^2^: 0.35, beta: 0.44 (95 % CI: 0.22–0.66), *p* < 0.001].

## Discussion

In our study, we recruited the representative group of patients with newly diagnosed autoimmune T1DM. In our catchment area, these patients are routinely hospitalized in a single center irrespective of the severity of their clinical presentation, therefore the fact of hospitalization is unlikely to introduce significant selection bias. In our study, we found significant association between serum adiponectin and HbA1c level, but not with glycaemia measured at diagnosis of diabetes. This finding may be explained by the greater stability of HbA1c level, which is also independent from transient confounders, as postprandial state, which significantly affect random glucose level. Similar association between elevated serum adiponectin concentration and HbA1c or hyperglycemia has been reported in other studies [1], although not equivocally [3]. Higher serum adiponectin concentration in patients with diabetic ketoacidosis at onset of T1DM may be attributed to the rise of adiponectin in conditions associated with increased catabolism and cachexia. However, association between DKA and serum adiponectin concentration was insignificant after adjustment for HbA1c. We also found a negative association between adiponectin and BMI at diagnosis and reported BMI before onset of symptoms of hyperglycaemia. The negative association between adiponectin and measures of insulin resistance was previously reported in patients with T1DM as in nondiabetic population [4]. Moreover, we noted the positive association between the degree of weight loss before T1DM diagnosis and adiponectin level which was not reported before. This may be explained either by greater relative weight loss occurring in overweight individuals or by the fact, that greater weight loss was associated with more severe presentation, significant catabolism and high prevalence of DKA. Higher adiponectin level in subjects positive for multiple islet autoantibodies may be also explained by more severe metabolic decompensation that was observed in these patients. Furthermore, we found negative association between serum C-peptide and adiponectin in men. Absence of similar association in women may be explained by the relatively small group of female participants in our study. This finding is similar to a study performed in adults with T1DM history of over 5 years [5]. Given the cross-sectional design of the study, we were unable to assess how body weight restoration and normalization of hyperglycemia on insulin treatment affect adiponectin value.

In our study, the association between HbA1c and adiponectin level remained significant after adjustment for the variables that reflect the severity of presentation at onset, namely presence of DKA and magnitude of weight loss before diagnosis. This suggests that chronic hyperglycemia is the major determinant of increased adiponectin concentration in patients with T1DM from the onset of the disease independently of the degree of metabolic decompensation and clinical indices of catabolism.
